# Differentiation of COVID-19 Pneumonitis and ICI Induced Pneumonitis

**DOI:** 10.3389/fonc.2020.577696

**Published:** 2020-10-29

**Authors:** Daphne W. Dumoulin, Hester A. Gietema, Marthe S. Paats, Lizza E. L. Hendriks, Robin Cornelissen

**Affiliations:** ^1^Department of Pulmonary Medicine, Erasmus Medical Center Cancer Institute, Rotterdam, Netherlands; ^2^Department of Radiology, Maastricht University Medical Center, Maastricht, Netherlands; ^3^GROW School for Oncology and Developmental Biology, Maastricht, Netherlands; ^4^Department of Pulmonary Medicine, Maastricht University Medical Center, Maastricht, Netherlands

**Keywords:** COVID-19, SARS-CoV-2, immune check inhibitor (ICI), co-rads, pneumonitis

## Abstract

Immune checkpoint inhibitors (ICI) have become the standard of care treatment for several tumor types. ICI-induced pneumonitis is a serious complication seen with treatment with these agents. Cancer has been reported to be one of the risk factors for severe coronavirus disease 2019 (COVID-19) caused by infection with severe acute respiratory syndrome coronavirus 2 (SARS-CoV-2), that has engulfed the world in the last couple of months. In patients with cancer treated with ICI who present at the emergency department with respiratory symptoms during the COVID-19 pandemic, correct diagnosis can be challenging. Symptoms and radiological features of ICI pneumonitis can be overlapping with those of COVID-19 related pneumonia. For the latter, dexamethasone and remdesivir have shown encouraging results, while vaccines are currently being evaluated in phase III trials. The mainstay of treatment in ICI pneumonitis is immunosuppressive therapy, as this is a potentially fatal adverse event. It has been speculated that immunosuppression may be associated with increased risk of progression to severe COVID-19, especially during the early stage of infection with SARS-CoV-2. Therefore, distinction between these two entities is warranted. We summarize the clinical, radiological features as well as additional investigations of both entities, and suggest a diagnostic algorithm for distinction between the two. This algorithm may be a supportive tool for clinicians to diagnose the underlying cause of the pneumonitis in patients treated with ICI during this COVID-19 pandemic.

## Introduction

Severe acute respiratory syndrome coronavirus 2 (SARS-CoV-2) is responsible for the coronavirus disease 2019 (COVID-19) pandemic that has infected 10 million people and caused half a million deaths as of June 30th, 2020 (1). Symptoms of COVID-19 can be similar to a common cold such as fever, coughing and shortness of breath. However, some patients experience a severe pneumonia, with acute respiratory distress syndrome (ARDS) and sometimes multi-organ failure. The in-hospital mortality is reported to be up to 25% (2). This is in contrast to the case fatality rate of COVID-19 in general, which was initially thought to be 15%, but in countries with rigorous testing appeared to be much lower at 0.3% (3).

Comorbidities were found in 77% of patients who died of severe COVID-19, with the majority being cardiopulmonary, liver or kidney disease, obesity, dementia, but also malignancy (2). Other factors associated with severe COVID-19 resulting in in-hospital mortality are increasing age and male sex. Cancer itself is a risk factor for infection with SARS-CoV-2, as patients with cancer commonly have an immunosuppressive status due to the cancer itself or due to the anticancer treatment (4). After adjusting for comorbidity, this risk of a severe outcome with COVID-19 seems to be higher for cancer patients. The highest risk of severe COVID-19 in cancer patients is seen in patients with lung or hematological cancer (5). In addition, patients with metastatic disease have a higher risk of severe outcome than patients with early stage disease. In the latter group, the outcome seems to be similar to non-cancer patients. Data regarding the impact of the specific anticancer therapy on the COVID-19 course was initially sparse. Recently, more insight was provided and anticancer therapy does not seem to influence the outcome of COVID-19 patients (6). Importantly, immune checkpoint inhibitors (ICI), standard of care treatment for several tumor types, do not appear to impact the severity of COVID-19 (7). However, one of the adverse events of ICI is ICI-induced pneumonitis and the clinical symptoms as well as the radiological findings can be overlapping with COVID-19 related pneumonia.

It can be a challenge to distinguish between ICI pneumonitis or COVID-19 pneumonia. ICI-induced pneumonitis is treated with systemic steroids and if necessary additional immunosuppressive drugs. Recently, the RECOVERY trial has shown that systemic corticosteroids reduce mortality in patients with COVID-19 needing invasive ventilation or supplemental oxygen therapy, but not in patient that do not require respiratory support. In addition, recently a short course of low dose corticosteroids proved to be safe in patients with non-severe COVID-19 (8). We should note, however, that while treatment with steroids may be reasonably safe based upon these reports, treatment with systemic steroids in the replication phase of respiratory viral infections was shown to be detrimental in the case of influenza virus (9). Discriminating between these two etiologies is of utmost importance to prevent patients being treated incorrectly, with an increased risk of either a severe course of ICI-induced pneumonitis or COVID-19 pneumonia. Here, we will discuss the pathophysiology, clinical symptoms, radiological features as well as additional investigations for both diseases. Lastly, we will discuss possible ways to help the clinician in establishing the correct diagnosis.

## ICI Pneumonitis

ICI-pneumonitis is defined as the development of dyspnea and/or other respiratory signs/symptoms in the presence of new infiltrates on chest imaging and in the absence of new respiratory infection (based on microbiology evaluation of either sputum of BAL fluid) in patients being treated with ICI (10). ICI-therapy derives its anti-tumor effect by blocking inhibitory pathways in the anti-tumor immune response. Blockade of the programmed death-1/programmed death-ligand 1(PD-1/PD-L1) pathway is a frequently used treatment in several malignancies. It can lead to a decrease in immune self-tolerance, which in turn may lead to a plethora of side effects in the form of inflammation of the organ(s) in question (11–13), one of which is pneumonitis (10).

The reported incidence for ICI pneumonitis is around 1–7% with higher incidences reported in patients treated with combination therapy (14, 15). However, outside of clinical trials the reported incidence appears strikingly higher and is up to 19% (15). The incidence of pneumonitis may also be related to tumor type as it seems to be higher in patients with non-small cell lung cancer than in patients with melanoma (16). The mortality rate of ICI-pneumonitis is higher compared with immune related adverse events associated with other organs and can approach 25% in some studies (16, 17).

### Clinical Manifestation

The most common symptoms of ICI pneumonitis are dyspnea and coughing. Chest pain can also occur, and sometimes patients present with fever and infectious causes need to be excluded (16). Physical examination can be normal, although in some patient Velcro crackles can be heard in the lungs. ICI pneumonitis can start from initiation of ICI therapy (18) and can even occur after discontinuarion of ICI treatment (19) with a median time to onset of 2.8 months (20). The onset can be acute, subacute or chronic. While some patients with ICI pneumonitis do not experience any symptoms (16), in some patients presenting with the most severe form of ICI pneumonitis, tachypnea, severe hypoxemia, and widespread alveolar infiltrates are often seen. This severe clinical entity is similar to the acute respiratory distress syndrome (ARDS).

### Laboratory Examination

In laboratory examination, routine blood tests may show elevated white blood cells and/or neutrophils, but normal blood counts can be present as well. Erythrocyte sedimentation and C-reactive protein are often elevated (16).

### Bronchoscopy

In ICI pneumonitis, bronchoscopy is mainly used to exclude infectious pneumonia. Using bronchoscopy, a deep sputum sample can be collected, which is reliable for elimination of the differential diagnosis of infection and guidance of anti-microbial therapy (21). In ICI pneumonitis, BAL fluid often show lymphocytic inflammation of alveoli with an increased proportion of lymphocytes in the cell count [normal lymphocyte count ≈11%, in ICI pneumonitis ≈37% (22, 23)]. Transbronchial biopsies during bronchoscopy can be considered and may be helpful to make the diagnosis (24).

### Imaging

The imaging modality of choice is high resolution computed tomography (HRCT). A wide spectrum of imaging manifestations in ICI–related pneumonitis has been reported including ground glass areas, consolidations, septal thickening, traction bronchiectasis and features of acute interstitial pneumonitis (AIP), such as consolidations and volume loss, dependent on severity of toxicity (20). Areas of ground glass and consolidation are most commonly reported, noted in 70–80% of cases (15), while septal thickening and traction bronchiectasis are only noted in 15–20% of cases.

**Figure 1:**
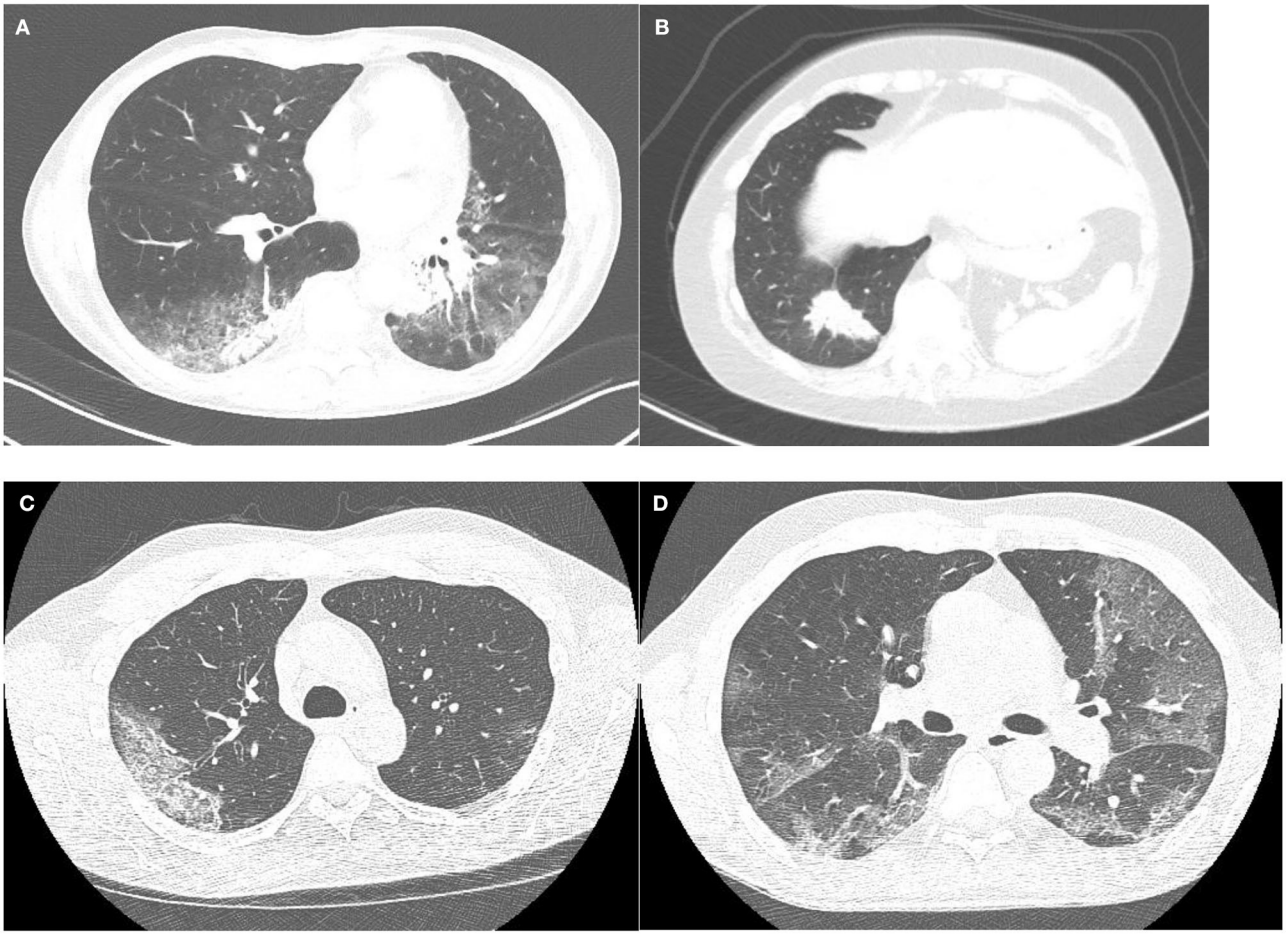
**(A,B)** A patient with ICI pneumonitis. Although ground glass opacities are present **(A)**, consolidations outside the areas of ground glass opacities are the dominant feature **(B)**, corresponding to a pattern of organizing pneumonia. **(C,D)** Two cases of RT-PCR proven COVID-19. One patient with typical well-demarcated slightly rounded subpleural ground glass opacities with thickened inter- and intralobular septa within this area (crazy paving): CO-RADS 5 **(C)**. The second patient shows well-demarcated, but not rounded, bilateral subpleural ground glass opacities with early thickening inter- and intralobular septa **(D)**. During the COVID-19 pandemic this case was judged as very highly suspicious (CO-RADS 5), but this case lacks confirmatory features.

Manifestations of ICI-pneumonitis are mostly multifocal, with peripheral involvement as the second most common; the lower lobes are most frequently involved (25). These manifestations mainly show patterns of interstitial lung diseases (Figures 1A,B).

The most commonly observed ICI pattern is organizing pneumonia (OP), present in 65% of ICI pneumonitis (10), showing bilateral patchy opacities with a peripheral or peribronchovascular predominance mainly located in the middle and lower parts of the lungs (24, 26). These opacities can be combined with ground glass opacities (GGO) and pulmonary nodules can also be seen (27). The second most described pattern in ICI-therapy is the non-specific interstitial pneumonia (NSIP) pattern, which is found in 15% of ICI pneumonitis (10, 24). NSIP shows GGOs and subpleural reticulation with a lower zone predominance and usually a fairly symmetric presentation. Hypersensitivity pneumonitis (HP) is a less common manifestation accounting for 10% of the ICI pneumonitis presenting with ill-defined centrilobular nodules, similar to the nodules seen in HP due to other triggers (10). The most severe form of ICI-pneumonitis is the AIP, occurring in 10% of ICI pneumonitis (28). AIP presents with diffuse GGO or consolidative opacities involving the majority and sometimes the entirety of the lungs. In addition, interlobular septal thickening and “crazy-paving” patterns (i.e., combination of GGO and reticulation) can be seen (27). Of note, these different patterns of ICI-related pneumonitis can coexist in the same patient, either simultaneously or successively in case of rechallenge with ICIs.

### Pathology

As in radiologic assessment, four accompanying key patterns of ICI pneumonitis can be recognized on pathologic examination; OP, NSIP, HP, and diffuse alveolar damage (DAD) (21).

OP affects distal bronchioles, respiratory bronchioles, alveolar ducts, and alveolar walls and is typically characterized by excessive proliferation of granulation tissue plugs in the distal airspaces with infiltration by plasma cells and lymphocytes (29). In NSIP, the findings on histological samples are fibrosis with inflammatory cell infiltration which is diffuse combined with uniform and diffuse thickening of alveolar walls (30). Histopathological examination of HP usually is characterized by poorly formed non-caseating granulomas (31). DAD is the histopathologic entity which is found in patients with AIP and is characterized by widespread injury of the alveoli that results in substantial capillary leak and non-cardiogenic pulmonary edema. Histopathologic appearance of DAD is similar to that of ARDS and is characterized by the thickening of the alveolar membranes, hyaline membrane deposition in addition to infiltration with inflammatory cells (28).

## COVID-19

SARS-CoV-2 is caused by an recently emerged RNA coronavirus related to SARS-CoV and Middle East respiratory syndrome coronavirus (MERS-CoV). Symptoms of SARS-CoV-2 infection are also similar to the other coronaviruses (32). Although cases have been described at all ages, the virus is more likely to lead to severe interstitial pneumonia in high-risk individuals, such as the elderly and those with comorbidities such as cancer. This can in turn lead to ARDS and multiorgan failure, which can prove to be fatal. Affected individuals can present themselves with different clinical symptoms, biochemical abnormalities and radiological findings (33, 34). In contrast to the seasonal flu, bacterial superinfection does not seem to play a major role in the fatality associated with SARS-COV-2.

### Clinical Manifestation

The clinical manifestation of COVID-19 can range from mild to severe (35). The most reported symptoms are fever, cough, dyspnea, myalgia or fatigue. Less commonly reported symptoms include headache, loss of smell and taste, abdominal pain, diarrhea, runny nose, and productive cough (35).

### Virology

Reverse transcriptase polymerase chain reaction (RT-PCR) can be performed on nasopharyngeal swabs, tracheal aspirate or BAL specimens. BAL specimens give the highest positive rates (93%), followed by sputum (72%), nasal swabs (63%), bronchoscopic brush (46%), and phanyngeal swabs (32%) (36). A negative test, therefore, does not exclude SARS-CoV-2 infection, especially in highly exposed persons and at the beginning of the infection. If only a swab was performed, it may be advisable to repeat the test or to collect a deeper respiratory tract sample, such as tracheal aspirate or BAL. Nevertheless, as the RT-PCR of SARS-CoV-2 RNA has a high specificity, but a limited sensitivity of around 77% (37), biochemical and radiological features might be useful in diagnosing COVID-19, especially in clinically highly suspect cases with initial negative PCR results (38) and for triage while awaiting RT-PCR results.

### Laboratory Examination

Laboratory examination shows significantly reduced lymphocyte counts in majority of patients and is correlated with the severity of disease (39). In addition, prolongation of prothrombin times (PT) and elevation of fibrinogen degradation products (FDPs) and D-dimers have been described. Soluble interleukin-2 receptor (sIL-2R) and interleukin-6 (IL-6) were significantly increased in patients with severe COVID-19. In all these measurements, critically ill patients had significantly higher/lower values (depending on tested laboratory parameter) than less severe cases (40). Serum ferritin levels are also elevated in severe cases of SARS-CoV-2 infection (40–42). Overall, laboratory examination can aid in establishing the diagnosis, but none of the blood tests are sensitive or specific to make the diagnosis of acute SARS-CoV-2 infection by themselves.

### Bronchoscopy

Little is known about the bronchoscopic findings in patients with COVID-19; as bronchoscopy is associated with an increased risk of infection with SARS-CoV-2 for the health care personnel, and is therefore not frequently performed. The (inter)national guidelines state the following (43): Bronchoscopy (flexible and rigid) for urgent or emergent reasons should be considered only if a lifesaving bronchoscopic intervention is deemed necessary. Indications include massive hemoptysis, benign or malignant severe airway stenosis, or suspicion of an alternative or secondary infectious etiology or malignant condition with resultant significant endobronchial obstruction. However, when caring for patients where the initial evaluations are not conclusive in making the distinction between COVID-19 and ICI-pneumonitis, bronchoscopy may prove to be useful to collect specimens needed to test SARS-CoV-2 *via* RT-PCR, given the higher positive rate of BAL when compared to nasopharyngeal swab. Of note, bronchoscopy must be performed while taking maximal precautions including personal protective equipment.

### Imaging

Early reports from Wuhan, China, described typical CT features seen in COVID-19 pneumonia and since the spread of the disease over Europe and other parts of the world, many publications from different countries have described the typical features seen on imaging (44–48).

Typical CT features of COVID-19 include bilateral subpleural areas of GGOs, mainly in the lower lung zones and are often extensive (Figures 1C,D). Features have been described to be dependent on disease duration with development of thickened intralobular septa in the areas of GGOs after 8–10 days of disease resulting in a crazy paving pattern. In those cases that progress to severe disease, areas of consolidation can develop, both in the pre-existent GGO areas but also in lung areas that were previously not involved. In the healing stage of disease features of organizing pneumonia are seen.

Although the typical hallmarks of COVID-19 related pneumonia on CT imaging are very rarely seen in other diseases, not all patients with COVID-19 present with these typical features. It has been described that about half of the patient population has a normal CT scan up to 48 h after start of symptoms (49). Early publications reported an extremely high sensitivity (up to 97%) for CT scanning in diagnosing COVID-19 (50), but with a low specificity of only 37%. Therefore, several standard reporting schemes were developed to improve communication about level of suspicion based on imaging features (51, 52). In the Netherlands, the COVID-19 reporting and data system (CO-RADS) was developed and used to grade the level of suspicion on COVID-19 based on CT features in patients with moderate to severe symptoms with good accuracy in predicting COVID-19 (52). This reporting system ranges from CO-RADS 0 (not interpretable) to CO-RADS 6 (RT-PCR proven SARS-CoV2) (52), for radiologists, the most relevant ranges are from CO-RADS 1 (no signs of infectious disease) to CO-RADS 5 (very high likelihood of COVID-19), with CO-RADS 3 being equivocal. CO-RADS 3 can represent early cases of COVID-19 with an unifocal ground glass opacity, but also cases with ground glass without the typical subpleural (including the mediastinal pleura and the fissures) location and less well-demarcated or diffuse ground glass, often noted in other viral pneumonias, but also seen in heart failure, drug induced pulmonary disease and focal fibrosis. NSIP pattern can be seen in healing disease, but is rare in the acute phase.

Several studies have reported on the high incidence of pulmonary emboli in patients with COVID-19 (53, 54). Therefore, it has been proposed to add pulmonary CTA in the diagnostic pathway for COVID-19 (55).

### Pathology

COVID-19 related ARDS causes pathological changes of DAD in the lungs. Recently, the longer term outcomes of ARDS have reported lung fibrosis as a possible outcome of COVID-19 ARDS (56). Fibrous lesions in fact may form during the healing of pulmonary chronic inflammation or proliferative diseases, with scar tissue gradually replacing cellular components (56).

Soon after the COVID-19 outbreak, multiple papers reported autopsy findings of pulmonary embolisms (57, 58). Coagulation dysfunction appears to be common in COVID-19, with the lungs from patients with COVID-19 showing distinctive vascular features, consisting of severe endothelial injury associated with the presence of intracellular virus and disrupted cell membranes. Histologic analysis of pulmonary vessels in patients with COVID-19 showed widespread thrombosis with microangiopathy (59). In a study with 184 patients with COVID-19 admitted to ICU, a 31% incidence of thrombotic complications was shown reinforcing the recommendation to strictly apply pharmacological thrombosis prophylaxis in all patients with COVID-19 admitted to the ICU (53). In addition, pulmonary embolisms should always be considered as a differential diagnosis, for which therapy guidelines are in place (60).

## Discussion

For patients with cancer receiving immunotherapy that have symptoms like dyspnea, fever and/or coughing, distinguishing between ICI pneumonitis or COVID-19 is challenging. However, discrimination between these two entities is essential to provide optimal care to the patient.

Misdiagnosis of COVID-19 with ICI pneumonitis can result in corticosteroid administration during the replication phase of the virus, the outcomes of which are currently unknown. In addition, treating a patient incorrectly for ICI pneumonitis instead of diagnosing COVID-19 could have negative consequences for the cancer treatment. On the other hand, not timely treating ICI pneumonitis can result in irreversible lung injury with a fulminant clinical course. Therefore, recognizing ICI pneumonitis is crucial, given the high mortality associated with this clinical entity.

Since clinical presentation of ICI pneumonitis and COVID-19 are similar and RT-PCR results can take up to 24 h and can be false negative, diagnostic tools such as lab results and imaging can be helpful for discrimination between both entities.

With regard to imaging, ICI pneumonitis and COVID-19 show overlapping features and discrimination on HRCT can be difficult. Both entities can show patchy GGOs and areas of consolidation, often with a fairly symmetrical distribution and a lower lobe and peripheral predominance. Several reporting systems have been developed to rate the level of suspicion on COVID-19 based on well-described features with CO-RADS being reported to have good interobserver agreement (52) in contrast to the reporting system proposed by the RSNA (61). These features include GGO areas with or without areas of consolidation, in lung regions close to the visceral pleural surfaces with a multifocal, bilateral distribution. Crazy paving pattern, rounded shape of the GGO areas and vessel dilatation are confirmatory features. CO-RADS categories 4–5 display a high to remarkably high likelihood of COVID-19 in a high prevalence situation with good to excellent accuracy (52). Not all cases of COVID-19 present with typical features and some patients show ground glass opacities without the typical distribution and without confirmatory features, fulfilling the criteria for CO-RADS 3 (i.e., equivocal). In this category an alternative cause of symptoms including severe dyspnea is often likely.

The hallmarks of ICI pneumonitis overlap with features of COVID-19. However, while a peripheral distribution is common in both entities, COVID-19 is also frequently located along the mediastinal pleural surfaces and the fissures. Crazy paving is frequently seen in COVID-10 patients who have symptoms for more than a week, while reticulation in ICI-pneumonitis is often seen concomitant with areas of ground glass, but not within the ground glass opacifications. Features of organizing pneumonia are common in ICI pneumonitis, but also seen in COVID-19. However, in COVID-19 these features are rarely seen as the main feature in contrast to cases of ICI pneumonitis. Furthermore, GGO is the major feature in early COVID-19, while features of organizing pneumonia are seen later in the course of disease. NSIP pattern is often seen in ICI pneumonitis, but rare in cases of acute COVID-19.

While both ICI pneumonitis and COVID-19 have typical features which makes the alternative diagnosis less likely, not all patients present with typical manifestations. In these cases, imaging is not able to discriminate between both entities. Fortunately, the number of CO-RADS 3 cases during the peak of the COVID-19 pandemic was low (52).

**Figure 2:**
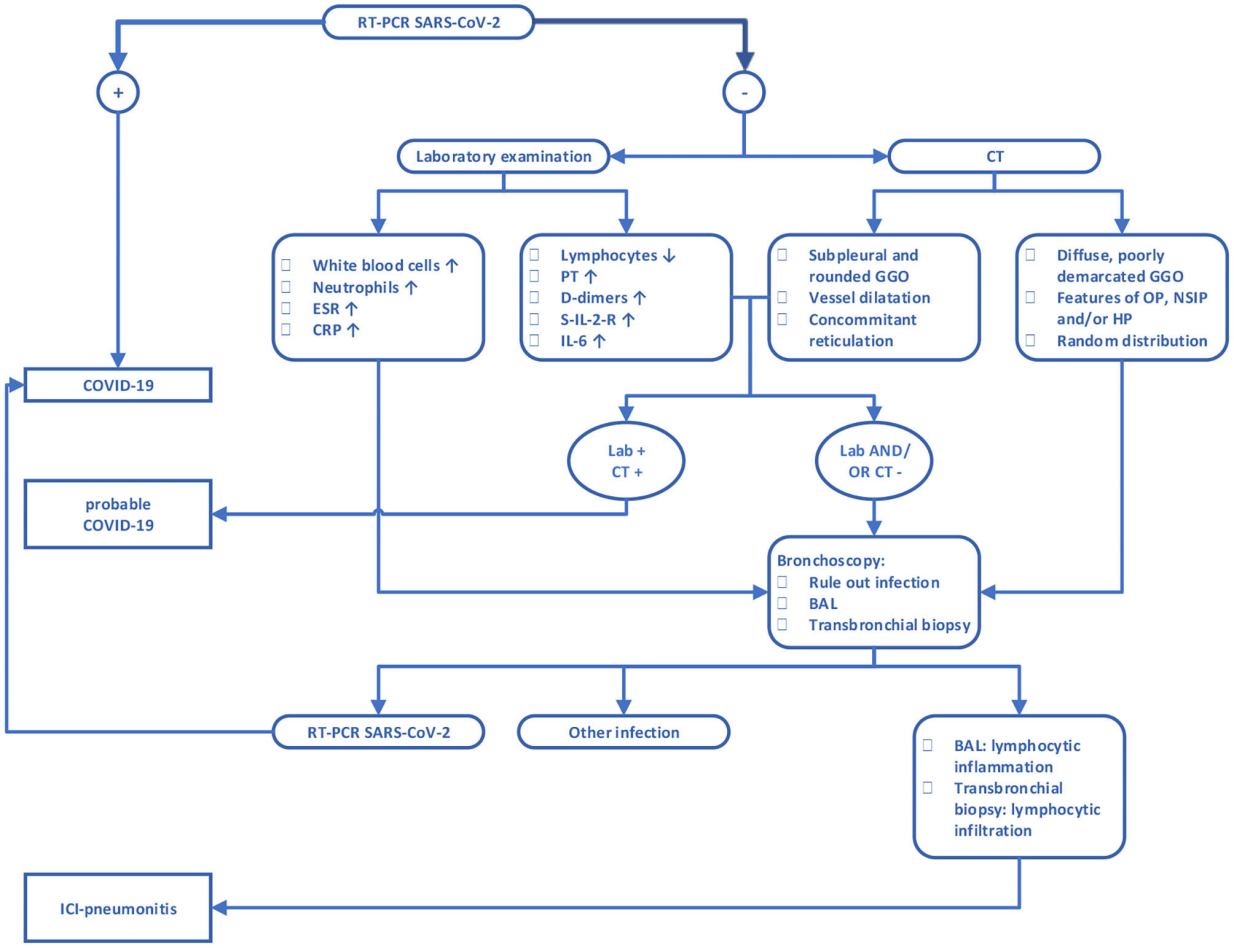
Diagnostic algorithm for discrimination between COVID-19 and ICI pneumonitis. CRP, C-reactive protein; ERS, erythrocyte sedimentation rate; PT, prothrombin time; S-IL-2-R, soluble interleukin 2 receptor; IL-6, interleukin 6; BAL, bronchoalveolar lavage; GGO, ground-glass opacity; OP, organizing pneumonia; NSIP, non-specific interstitial pneumonia; HP, hypersensitivity pneumonitis. For features of OP, NSIP, and HP we refer to the accompanying text.

Given the mediocre sensitivity of the RT-PCR, we propose a diagnostic algorithm in order to assist clinicians to discriminate between COVID-19 and ICI pneumonitis based on laboratory, radiographical and pathological parameters, as described in Figure 2. First step is RT-PCR, which can confirm the diagnosis of COVID-19. However, due to the possibility of a false negative RT-PCR result, COVID-19 is not completely ruled out as a possible diagnosis. In those cases, laboratory examination and imaging features can be helpful in selecting patients who are still likely to have COVID-19 despite the negative RT-PCR. In addition, they may aid in determining the probability of ICI pneumonitis and may suggest need to perform further workup such as bronchoscopy. When the correct diagnosis has been made, the underlying cause should be managed. While ICI pneumonitis usually responds to treatment with corticosteroids, some patients are refractory to this treatment. Moreover, there may be suspicion for concurrent COVID-19 and ICI pneumonitis in some patients, despite following the suggested algorithm. In these patients, corticosteroids should be used with precaution.

## Conclusion

Discriminating between COVID-19 and ICI pneumonitis can be a challenging task for clinicians with any level of experience. The sensitivity of RT-PCR is low, and the consequences of wrong interpretation can be too severe, to fully trust a negative result in clinically suspicious cases. Therefore, additional parameters must be considered when assessing patients treated with ICI and a suspicion of COVID-19. Based on imaging, lab values, RT-PCR and if necessary bronchoscopy with BAL, we propose an algorithm to aid clinicians in daily practice to establish the right diagnosis leading to the initiation of correct treatment.

## Author Contributions

All authors contributed to the design and writing of the manuscript.

## Conflict of Interest

DD reports speakers fees from Roche, Pfizer, MSD, AstraZeneca, Novartis, BMS, and all outside the submitted work. MP reports honoraria for advisory boards from Bayer, Takeda, Lilly, Roche, and all outside the submitted work. LH reports honoraria for advisory boards from MSD, Boehringer, Pfizer, BMS, Eli Lilly, Takeda, Roche, speakers fees from Roche and MSD, and all outside the submitted work. RC reports honoraria for advisory boards from MSD and Roche, speakers fees from Roche, Pfizer, BMS, and all outside the submitted work. The remaining author declares that the research was conducted in the absence of any commercial or financial relationships that could be construed as a potential conflict of interest.

## References

[1] COVID-19 Map Johns Hopkins Coronavirus Resour Cent. (2020). Available online at: https://coronavirus.jhu.edu/map.html (accessed June 28, 2020).

[2] Docherty AB Harrison EM Green CA Hardwick HE Pius R Norman L. Features of 20 133 UK patients in hospital with covid-19 using the ISARIC WHO Clinical Characterisation Protocol: prospective observational cohort study. BMJ. (2020) 369:m1985. 10.1136/bmj.m1985 32444460 PMC7243036

[3] Rajgor DD Lee MH Archuleta S Bagdasarian N Quek SC. The many estimates of the COVID-19 case fatality rate. Lancet Infect Dis. (2020) 20:776–7. 10.1016/S1473-3099(20)30244-9 32224313 PMC7270047

[4] Liang W Guan W Chen R Wang W Li J Xu K. Cancer patients in SARS-CoV-2 infection: a nationwide analysis in China. Lancet Oncol. (2020) 21:335–7. 10.1016/S1470-2045(20)30096-6 32066541 PMC7159000

[5] Dai M Liu D Liu M Zhou F Li G Chen Z. Patients with cancer appear more vulnerable to SARS-CoV-2: a multicenter study during the COVID-19 outbreak. Cancer Discov. (2020) 10:783–91. 10.1158/2159-8290.CD-20-0422 32345594 PMC7309152

[6] Garassino MC Whisenant JG Huang L-C Trama A Torri V Agustoni F. COVID-19 in patients with thoracic malignancies (TERAVOLT): first results of an international, registry-based, cohort study. Lancet Oncol. (2020) 21:914–22. 10.1016/S1470-2045(20)30314-4 32539942 PMC7292610

[7] Luo J Rizvi H Egger JV Preeshagul IR Wolchok JD Hellmann MD. Impact of PD-1 blockade on severity of COVID-19 in patients with lung cancers. Cancer Discov. (2020) 10:1121–8. 10.1158/2159-8290.CD-20-0596 32398243 PMC7416461

[8] Hu Z Lv Y Xu C Sun W Chen W Peng Z. Clinical use of short-course and low-dose corticosteroids in patients with non-severe COVID-19 during pneumonia progression. Front Public Health. (2020) 8:355. 10.3389/fpubh.2020.00355 32719766 PMC7349005

[9] Yang J-W Fan L-C Miao X-Y Mao B Li M-H Lu H-W. Corticosteroids for the treatment of human infection with influenza virus: a systematic review and meta-analysis. Clin Microbiol Infect. (2015) 21:956–63. 10.1016/j.cmi.2015.06.022 26123860

[10] Suresh K Naidoo J Lin CT Danoff S. Immune checkpoint immunotherapy for non-small cell lung cancer: benefits and pulmonary toxicities. Chest. (2018) 154:1416–23. 10.1016/j.chest.2018.08.1048 30189190 PMC6335259

[11] Dumoulin DW Visser S Cornelissen R van Gelder T Vansteenkiste J von der Thusen J. Renal toxicity from pemetrexed and pembrolizumab in the era of combination therapy in patients with metastatic non-squamous cell NSCLC. J Thorac Oncol Off Publ Int Assoc Study Lung Cancer. (2020) 15:1472–83. 10.1016/j.jtho.2020.04.021 32360753

[12] Prieux-Klotz C Dior M Damotte D Dreanic J Brieau B Brezault C. Immune checkpoint inhibitor-induced colitis: diagnosis and management. Target Oncol. (2017) 12:301–8. 10.1007/s11523-017-0495-4 28540478

[13] Yshii LM Hohlfeld R Liblau RS. Inflammatory CNS disease caused by immune checkpoint inhibitors: status and perspectives. Nat Rev Neurol. (2017) 13:755–63. 10.1038/nrneurol.2017.144 29104289

[14] Su Q Zhu EC Wu J-B Li T Hou Y-L Wang D-Y. Risk of pneumonitis and pneumonia associated with immune checkpoint inhibitors for solid tumors: a systematic review and meta-analysis. Front Immunol. (2019) 10:108. 10.3389/fimmu.2019.00108 30778352 PMC6369169

[15] Suresh K Voong KR Shankar B Forde PM Ettinger DS Marrone KA. Pneumonitis in non–small cell lung cancer patients receiving immune checkpoint immunotherapy: incidence and risk factors. J Thorac Oncol. (2018) 13:1930–9. 10.1016/j.jtho.2018.08.2035 30267842

[16] Wang H Guo X Zhou J Li Y Duan L Si X. Clinical diagnosis and treatment of immune checkpoint inhibitor-associated pneumonitis. Thorac Cancer. (2020) 11:191–7. 10.1111/1759-7714.13240 31762218 PMC6938759

[17] Tone M Izumo T Awano N Kuse N Inomata M Jo T. High mortality and poor treatment efficacy of immune checkpoint inhibitors in patients with severe grade checkpoint inhibitor pneumonitis in non-small cell lung cancer. Thorac Cancer. (2019) 10:2006–12. 10.1111/1759-7714.13187 31482678 PMC6775002

[18] Tonk EHJ van Lindert ASR Verhoeff JJC Suijkerbuijk KPM. Acute-onset pneumonitis while administering the first dose of durvalumab. Case Rep Oncol. (2019) 12:621–4. 10.1159/000502202 31543778 PMC6738224

[19] Asher N Marom EM Ben-Betzalel G Baruch EN Steinberg-Silman Y Schachter J. Recurrent pneumonitis in patients with melanoma treated with immune checkpoint inhibitors. Oncologist. (2019) 24:640–7. 10.1634/theoncologist.2018-0352 30777894 PMC6516115

[20] Naidoo J Wang X Woo KM Iyriboz T Halpenny D Cunningham J. Pneumonitis in patients treated with anti-programmed death-1/programmed death ligand 1 therapy. J Clin Oncol Off J Am Soc Clin Oncol. (2017) 35:709–17. 10.1200/JCO.2016.68.2005 27646942 PMC5559901

[21] Zhong L Altan M Shannon VR Sheshadri A. Immune-related adverse events: pneumonitis. Immunotherapy. (2020) 1244:255–69. 10.1007/978-3-030-41008-7_13 32301020 PMC7161534

[22] Heron M Grutters JC ten Dam-Molenkamp KM Hijdra D van Heugten-Roeling A Claessen AME. Bronchoalveolar lavage cell pattern from healthy human lung. Clin Exp Immunol. (2012) 167:523–31. 10.1111/j.1365-2249.2011.04529.x 22288596 PMC3374285

[23] Suzuki K Yanagihara T Matsumoto K Kusaba H Yamauchi T Ikematsu Y. Immune-checkpoint profiles for T cells in bronchoalveolar lavage fluid of patients with immune-checkpoint inhibitor-related interstitial lung disease. Int Immunol. (2020) 32:547–57. 10.1093/intimm/dxaa022 32253426

[24] Delaunay M Cadranel J Lusque A Meyer N Gounant V Moro-Sibilot D Michot J-M Raimbourg J Girard N Guisier F. Immune-checkpoint inhibitors associated with interstitial lung disease in cancer patients. Eur Respir J. (2017) 50:28798088 10.1183/13993003.00050-2017

[25] Nishino M Ramaiya NH Awad MM Sholl LM Maattala JA Taibi M. PD-1 inhibitor–related pneumonitis in advanced cancer patients: radiographic patterns and clinical course. Clin Cancer Res. (2016) 22:6051–60. 10.1158/1078-0432.CCR-16-1320 27535979 PMC5161686

[26] Nishino M Hatabu H Sholl LM Ramaiya NH. Thoracic complications of precision cancer therapies: a practical guide for radiologists in the new era of cancer care. Radiogr Rev Publ Radiol Soc N Am Inc. (2017) 37:1371–87. 10.1148/rg.2017170015 28898185 PMC5621730

[27] Kalisz KR Ramaiya NH Laukamp KR Gupta A. Immune checkpoint inhibitor therapy–related pneumonitis: patterns and management. RadioGraphics. (2019) 39:1923–37. 10.1148/rg.2019190036 31584861

[28] Larsen BT Chae JM Dixit AS Hartman TE Peikert T Roden AC. Clinical and histopathologic features of immune checkpoint inhibitor-related pneumonitis. Am J Surg Pathol. (2019) 43:1331–40. 10.1097/PAS.0000000000001298 31162288

[29] Cornelissen R Senan S Antonisse IE Liem H Tan YKY Rudolphus A. Bronchiolitis obliterans organizing pneumonia (BOOP) after thoracic radiotherapy for breast carcinoma. Radiat Oncol Lond Engl. (2007) 2:2. 10.1186/1748-717X-2-2 17201913 PMC1780052

[30] Travis WD Hunninghake G King TE Lynch DA Colby TV Galvin JR. Idiopathic non-specific interstitial pneumonia: report of an American Thoracic Society project. Am J Respir Crit Care Med. (2008) 177:1338–47. 10.1164/rccm.200611-1685OC 18388353

[31] Selman M Pardo A King TE. Hypersensitivity pneumonitis: insights in diagnosis and pathobiology. Am J Respir Crit Care Med. (2012) 186:314–24. 10.1164/rccm.201203-0513CI 22679012

[32] Wu D Wu T Liu Q Yang Z. The SARS-CoV-2 outbreak: what we know. Int J Infect Dis IJID Off Publ Int Soc Infect Dis. (2020) 94:44–8. 10.1016/j.ijid.2020.03.004 32171952 PMC7102543

[33] Pascarella G Strumia A Piliego C Bruno F Del Buono R Costa F. COVID-19 diagnosis and management: a comprehensive review. J Intern Med. (2020) 288:192–206. 10.1111/joim.13091 32348588 PMC7267177

[34] Liu K Chen Y Lin R Han K. Clinical features of COVID-19 in elderly patients: a comparison with young and middle-aged patients. J Infect. (2020) 80:e14–8. 10.1016/j.jinf.2020.03.005 32171866 PMC7102640

[35] Adhikari SP Meng S Wu Y-J Mao Y-P Ye R-X Wang Q-Z. Epidemiology, causes, clinical manifestation and diagnosis, prevention and control of coronavirus disease (COVID-19) during the early outbreak period: a scoping review. Infect Dis Poverty. (2020) 9:29. 10.1186/s40249-020-00646-x 32183901 PMC7079521

[36] Wang W Xu Y Gao R Lu R Han K Wu G. Detection of SARS-CoV-2 in different types of clinical specimens. JAMA. (2020) 323:1843–4. 10.1001/jama.2020.3786 32159775 PMC7066521

[37] He J-L Luo L Luo Z-D Lyu J-X Ng M-Y Shen X-P. Diagnostic performance between CT and initial real-time RT-PCR for clinically suspected 2019 coronavirus disease (COVID-19) patients outside Wuhan, China. Respir Med. (2020) 168:105980. 10.1016/j.rmed.2020.105980 32364959 PMC7172864

[38] Fang Y Zhang H Xie J Lin M Ying L Pang P. Sensitivity of chest CT for COVID-19: comparison to RT-PCR. Radiology. (2020) 296:E115–7. 10.1148/radiol.2020200432 32073353 PMC7233365

[39] Tavakolpour S Rakhshandehroo T Wei EX Rashidian M. Lymphopenia during the COVID-19 infection: what it shows and what can be learned. Immunol Lett. (2020) 225:31–2. 10.1016/j.imlet.2020.06.013 32569607 PMC7305732

[40] Li Y Hu Y Yu J Ma T Retrospective analysis of laboratory testing in 54 patients with severe- or critical-type 2019 novel coronavirus pneumonia. Lab Investig J Tech Methods Pathol. (2020) 100:794–800. 10.1038/s41374-020-0431-6 PMC7184820 32341519

[41] Guan W-J Ni Z-Y Hu Y Liang W-H Ou C-Q He J-X. Clinical characteristics of coronavirus disease 2019 in China. N Engl J Med. (2020) 382:1708–20. 10.1056/NEJMoa2002032 32109013 PMC7092819

[42] Terpos E Ntanasis-Stathopoulos I Elalamy I Kastritis E Sergentanis TN Politou M. Hematological findings and complications of COVID-19. Am J Hematol. (2020) 95:834–47. 10.1002/ajh.25829 32282949 PMC7262337

[43] Wahidi MM Lamb C Murgu S Musani A Shojaee S Sachdeva A American Association for Bronchology and Interventional Pulmonology (AABIP) statement on the use of bronchoscopy and respiratory specimen collection in patients with suspected or confirmed COVID-19 infection. J Bronchol Interv Pulmonol. (2020) 27:e52–4. 10.1097/LBR.0000000000000681 PMC7141581 32195687

[44] Wang J Xu Z Wang J Feng R An Y Ao W. CT characteristics of patients infected with 2019 novel coronavirus: association with clinical type. Clin Radiol. (2020) 75:408–14. 10.1016/j.crad.2020.04.001 32327229 PMC7138387

[45] Zhou S Wang Y Zhu T Xia L. CT features of coronavirus disease 2019 (COVID-19) pneumonia in 62 patients in Wuhan, China. AJR Am J Roentgenol. (2020) 214:1287–94. 10.2214/AJR.20.22975 32134681

[46] Pan Y Guan H Zhou S Wang Y Li Q Zhu T. Initial CT findings and temporal changes in patients with the novel coronavirus pneumonia (2019-nCoV): a study of 63 patients in Wuhan, China. Eur Radiol. (2020) 30:3306–9. 10.1007/s00330-020-06731-x 32055945 PMC7087663

[47] Chung M Bernheim A Mei X Zhang N Huang M Zeng X. CT imaging features of 2019 novel coronavirus (2019-nCoV). Radiology. (2020) 295:202–7. 10.1148/radiol.2020200230 32017661 PMC7194022

[48] Caruso D Zerunian M Polici M Pucciarelli F Polidori T Rucci C. Chest CT features of COVID-19 in Rome, Italy. Radiology. (2020) 296:E79–85. 10.1148/radiol.2020201237 32243238 PMC7194020

[49] Zhou S Zhu T Wang Y Xia L. Imaging features and evolution on CT in 100 COVID-19 pneumonia patients in Wuhan, China. Eur Radiol. (2020) 30:1–9. 10.1007/s00330-020-06879-6 32367418 PMC7197364

[50] Bai HX Hsieh B Xiong Z Halsey K Choi JW Tran TML. Performance of radiologists in differentiating COVID-19 from non-COVID-19 viral pneumonia at chest CT. Radiology. (2020) 296:E46–54. 10.1148/radiol.2020200823 32155105 PMC7233414

[51] Simpson S Kay FU Abbara S Bhalla S Chung JH Chung M. Radiological society of North America expert consensus statement on reporting chest CT findings related to COVID-19. Endorsed by the society of thoracic radiology, the American College of Radiology, and RSNA—Secondary Publication. J Thorac Imaging. (2020) 35:219–27. 10.1097/RTI.0000000000000524 32324653 PMC7255403

[52] Prokop M van Everdingen W van Rees Vellinga T Quarles van Ufford H Stöger L Beenen L. CO-RADS: a categorical CT assessment scheme for patients suspected of having COVID-19—definition and evaluation. Radiology. (2020) 296:E97–104. 10.1148/radiol.2020201473 32339082 PMC7233402

[53] Klok FA Kruip MJHA van der Meer NJM Arbous MS Gommers DaMPJ Kant KM. Incidence of thrombotic complications in critically ill ICU patients with COVID-19. Thromb Res. (2020) 191:145–7. 10.1016/j.thromres.2020.04.013 32291094 PMC7146714

[54] Poyiadji N Cormier P Patel PY Hadied MO Bhargava P Khanna K. Acute pulmonary embolism and COVID-19. Radiology. (2020). 10.1148/radiol.2020201955. [Epub ahead of print]. 32407256 PMC7706099

[55] Revel M-P Parkar AP Prosch H Silva M Sverzellati N Gleeson F. COVID-19 patients and the radiology department—advice from the European Society of Radiology (ESR) and the European Society of Thoracic Imaging (ESTI). Eur Radiol. (2020) 30:4903–9. 10.1007/s00330-020-06865-y 32314058 PMC7170031

[56] Gibson PG Qin L Puah SH. COVID-19 acute respiratory distress syndrome (ARDS): clinical features and differences from typical pre-COVID-19 ARDS. Med J Aust. (2020) 213:54–6.e1. 10.5694/mja2.50674 32572965 PMC7361309

[57] Wichmann D Sperhake J-P Lütgehetmann M Steurer S Edler C Heinemann A. Autopsy findings and venous thromboembolism in patients with COVID-19. Ann Intern Med. (2020) 173:268–77. 10.7326/M20-2003 32374815 PMC7240772

[58] Menter T Haslbauer JD Nienhold R Savic S Hopfer H Deigendesch N. Postmortem examination of COVID-19 patients reveals diffuse alveolar damage with severe capillary congestion and variegated findings in lungs and other organs suggesting vascular dysfunction. Histopathology. (2020) 77:198–209. 10.1111/his.14134 32364264 PMC7496150

[59] Ackermann M Verleden SE Kuehnel M Haverich A Welte T Laenger F. Pulmonary vascular endothelialitis, thrombosis, and angiogenesis in Covid-19. N Engl J Med. (2020) 383:120–8. 10.1056/NEJMoa2015432 32437596 PMC7412750

[60] Bikdeli B Madhavan MV Jimenez D Chuich T Dreyfus I Driggin E. COVID-19 and thrombotic or thromboembolic disease: implications for prevention, antithrombotic therapy, and follow-up: JACC state-of-the-art review. J Am Coll Cardiol. (2020) 75:2950–73. 10.1016/j.jacc.2020.04.031 32311448 PMC7164881

[61] de Jaegere TMH Krdzalic J Fasen BACM Kwee RM Radiological Society of North America Chest CT Classification System for reporting COVID-19 pneumonia: interobserver variability and correlation with RT-PCR. Radiol Cardiothorac Imaging. (2020) 2:e200213 10.1148/ryct.2020200213 PMC7294823 33778589

